# Multi-Channel Microfluidic Biosensor Platform Applied for Online Monitoring and Screening of Biofilm Formation and Activity

**DOI:** 10.1371/journal.pone.0117300

**Published:** 2015-02-23

**Authors:** Julia Bruchmann, Kai Sachsenheimer, Bastian E. Rapp, Thomas Schwartz

**Affiliations:** 1 Institute of Functional Interfaces (IFG), Karlsruhe Institute of Technology (KIT), Eggenstein-Leopoldshafen, Germany; 2 Institute of Microstructure Technology (IMT), Karlsruhe Institute of Technology (KIT), Eggenstein-Leopoldshafen, Germany; Université d'Auvergne Clermont 1, FRANCE

## Abstract

Bacterial colonization of surfaces and interfaces has a major impact on various areas including biotechnology, medicine, food industries, and water technologies. In most of these areas biofilm development has a strong impact on hygiene situations, product quality, and process efficacies. In consequence, biofilm manipulation and prevention is a fundamental issue to avoid adverse impacts. For such scenario online, non-destructive biofilm monitoring systems become important in many technical and industrial applications. This study reports such a system in form of a microfluidic sensor platform based on the combination of electrical impedance spectroscopy and amperometric current measurement, which allows sensitive online measurement of biofilm formation and activity. A total number of 12 parallel fluidic channels enable real-time online screening of various biofilms formed by different Pseudomonas aeruginosa and Stenotrophomonas maltophilia strains and complex mixed population biofilms. Experiments using disinfectant and antibiofilm reagents demonstrate that the biofilm sensor is able to discriminate between inactivation/killing of bacteria and destabilization of biofilm structures. The impedance and amperometric sensor data demonstrated the high dynamics of biofilms as a consequence of distinct responses to chemical treatment strategies. Gene expression of flagellar and fimbrial genes of biofilms grown inside the microfluidic system supported the detected biofilm growth kinetics. Thus, the presented biosensor platform is a qualified tool for assessing biofilm formation in specific environments and for evaluating the effectiveness of antibiofilm treatment strategies.

## Introduction

Biofilm formation on technical surfaces is still one of the most challenging problems regarding biofouling in aqueous systems. Technical and industrial applications provide various surfaces as substratum for biofilm adhesion. Here, biofilms, e.g. in drinking water environments, are known as potential source for the spread of hygienically relevant bacteria [1]. Often, biofouling in industrial processes is detected by product contamination or a decline in process performance. Early-on warning systems could reduce defiled production and reduce the substantial cleaning costs of several billions US$ each year worldwide [2]. Efficient surveillance of biofouling requires appropriate online detection systems which monitor biofilm formation and monitor on-demand cleaning strategies. However, early-on warning and real-time monitoring systems are still rare and therefore require development of new online non-destructive sensitive detection methods.

Generally, biofilms are composed of attached microbial cell communities. The attachment of bacteria to surfaces is mediated by expression and activity of flagella, fimbria and type IV pili in Gram-negative bacteria [3] or adhesion proteins in Gram-positive bacteria [4]. The attached microorganisms are forming microcolonies on surfaces, where the bacteria are embedded in extracellular polymeric substances (EPS) [5]. These EPS consisting of polysaccharides, proteins and extracellular DNA (eDNA) provide protection and increased resistance towards mechanical, physical, and chemical treatment [6]. During maturation the biofilms built up a three-dimensional structure with intermolecular connections providing high biofilm stability. Diffusion inhibition, cell-cell communication, gene transfer, and high diversity among subpopulations give a survival benefit to the biofilms, which makes them hard to completely eradicate by conventional methods based on biocide probing [7]. Novel strategies for anti-biofouling treatment therefore focus on destabilization of the EPS matrix, enabling penetration of antimicrobials into the biofilm [8]. By weakening of the biofilm’s structure the removal from the attached surfaces is facilitated.

Biofilm monitoring techniques are therefore fundamental for understanding and, in the end, controlling biofilm dynamics which is a necessary prerequisite to prevent or successfully manipulate biofilm formation. A huge selection of different methods for biofilm characterization is available nowadays, differing in properties such as scale, handling time, sensitivity, and the detection method employed. A lot of techniques are destructive endpoint diagnosis, requiring removal of biofilms from the growth reactor [9,10] which renders them unsuitable for online monitoring. Fixation and staining methods leaving the biofilm intact are mostly limited to one time point diagnosis and are often time consuming. To overcome these hurdles continuous non-destructive online monitoring methods are required.

In the last decades techniques providing continuous biofilm monitoring were developed based on different detection methods. Spectroscopic techniques detecting fluorescence, refraction transmission and scattering are online and fast but limited to fluids with low turbidity [11,12]. Also gravimetric detection techniques based on quartz crystal microbalance have been described and used for sensitive online monitoring of conditioning films on surfaces [13] as well as for long-term biofilm studies [14] in the last years. This technique has limitations for early-warning approaches and does not allow discrimination between biomass accumulation and activity. Alternative detection techniques such as surface plasmon resonance [15] and surface acoustic waves [16] have also been used for non-destructive biofilm monitoring with similar limitations. Increasingly the advantages of microfluidic scale reactors are being recognized. Providing precise control of the microenvironment with highly reproducible conditions, microfluidics enables lab-on-chip analysis at small volumes [17].

Electrical impedance spectroscopy (EIS) based sensors measuring biofilm formation were previously used in various compositions such as petri dishes for early detection of biofilm formation [18], in a modified CDC reactor simulating *in vivo* flow conditions [19], as well as to detect biomass transfer on rotating disc electrodes [20]. However, these sensors focused purely on biomass detection. Critical parameters such as activity in combination with attached biomass are important for the evaluation of cleaning procedures and to gain deeper insight into biofilm formation dynamics. However, these parameters are not accessible with these systems. Moreover, so far established online biosensors are lacking the possibility for parallel multichannel analysis which is a prerequisite for high-throughput characterization, e.g., of antibiofilm or cleaning strategies.

In a proof of principal study we have previously described EIS as a suitable technique for online monitoring of biofilm formation [21]. EIS as detection method was combined with amperometric current measurement using a microfluidic setup resulting in a flexible microfluidic-based electrochemical characterization platform. Based on this study we developed a multichannel system which we describe in this work.

This platform is a microfluidic biosensor platform combining EIS and amperometric current measurement on a 12 channel parallel platform for the online detection and monitoring of bacterial biofilm formation and respiratory activity. Biomass increase is measured by inhibition of charge transfer at the measurement electrodes during biofilm formation. The measured amperometric current correlates with the respiratory activity of the biofilms [22]. Both parameters allow characterizing the overall biomass and its activity in real-time. In addition to this real-time monitoring, we describe the evaluation of genetic parameters of biofilms grown within the sensor platform which enables identification of regulatory networks or molecular responses. This combination results in a powerful sensor platform for collateral screening of biofilm formation potential including biofilm genetics as well as for parallelized screening for biofilm modulating substances, providing insights into biofilm dynamics, destabilization, and inactivation.

## Material and Methods

### Biosensor design and manufacturing

**Fig 1:**
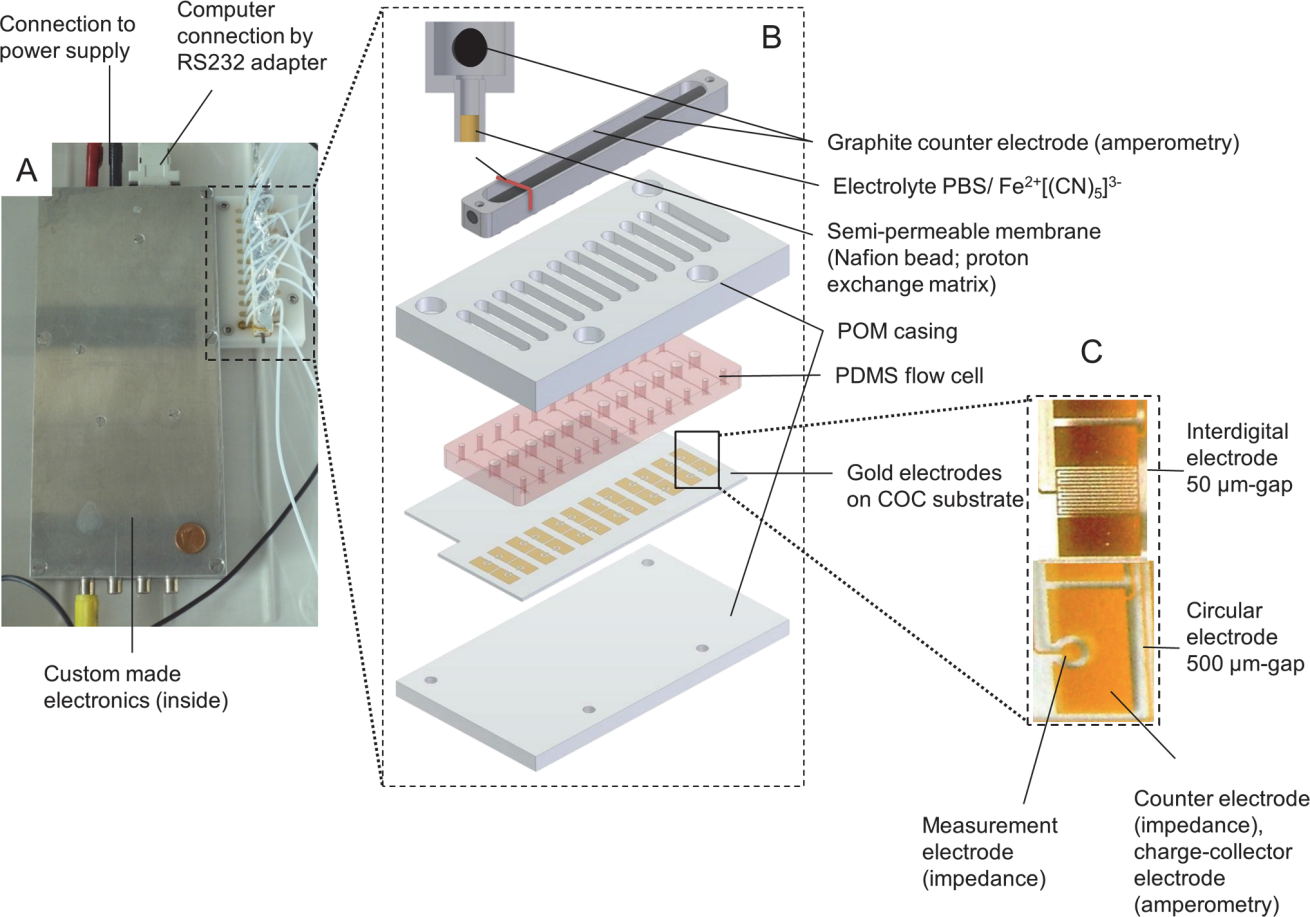
Sensor system. The sensor system consists of a 12-flow channel module connectable to a custom-made electronics. The measurement electronics is designed with four ports for expandable connection of further 12-flow channel units (A). A 12-flow channel unit consists of an amperometric counter electrode and the fluidically independent microfluidic flow channels sealed by a substrate with planar gold-electrode structures (B). Different electrode designs were used depending on the application including circular electrodes with a gap of 500 μm and interdigitated electrodes with gaps of 15–100 μm (C).

**Fig 2:**
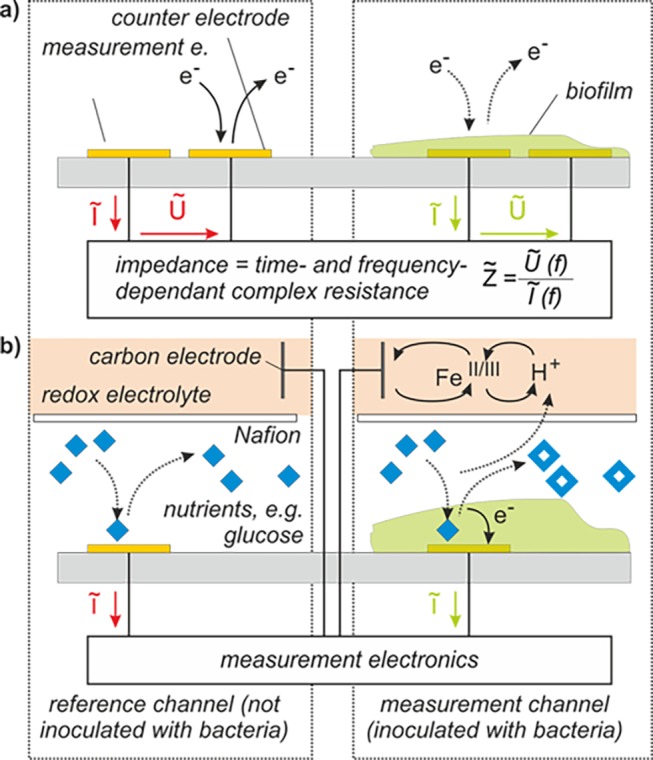
Schematic of the measurement setup. The system operates in differential measurement mode using reference channels (bacteria-free) and measurement channels (probed with bacteria). The evaluated sensor signals are differential signals formed between two electrodes from either category. This differential measurement allows reducing sensor drift due to ambient influences (temperature, etc.). The schematic depicts one electrode in a reference channel (bacteria free) and an electrode in a measurement channel (with bacteria). Each channel has a total of two electrodes (each consisting of a measurement electrode and a counter electrode) for electrochemical impedance spectroscopy (EIS). Each microfluidic channel is additionally equipped with two amperometric electrodes, which consist of a chamber filled with electrolyte solution that is fluidically separated from the microfluidic channel by a proton exchange membrane (PEM). A carbon rod is inserted in this chamber and serves as the cathode, the EIS counter electrodes serve as the anode. The biofilm respiratory activity can be measured directly by measuring the current created during respiratory activity via the electrons collected by the cathode. The EIS electronic module is disconnected in amperometric measure mode and the amperometric electronic module is disconnected in EIS measure mode to avoid interferences.

The setup of the biosensor system can be seen in Fig. 1. It consists of a 12-flow channel unit, wherein the biofilm is formed. This module can be connected to the measurement electronics via spring-loaded circuit board connectors. The electronics is designed with 4 connector ports allowing modular system extension. Each flow channel contains two independent EIS electrodes and two amperometric activity electrodes. The measurement principle is depicted in Fig. 2 according to our previous study [21]. The flow cells are made of polydimethylsiloxane (PDMS; height: 0.5 mm, width: 4 mm, lengths 18.9 mm) by casting from a molding tool created by CNC milling in polymethylmethacrylate (PMMA). The PDMS flow cell is pressed onto a cyclic olefin copolymer (COC) substrate on top of which the gold electrodes are located. The latter are created by means of photolithography and gold etching as described in our previous study [21]. Briefly, a thin layer of gold is sputtered onto the substrates and coated with a positive-type photoresist (AZ4562, purchased from MicroChemicals GmbH) and structured via photomasks. Different photomask designs were fabricated in order to test different electrode designs. Circular electrodes with gaps of 500 μm between measurement and counter electrode as well as interdigitated electrodes with gaps ranging between 15 μm and 100 μm were tested. The photoresist was developed in a developer solution of AZ351B (purchased from MicroChemicals GmbH, diluted 1:4 v/v with bidistilled water) removing the exposed areas of the photoresist prior to gold etching in solution (1:4 m/m solution of iodine/potassium iodine in bidistilled water) and stripping of the resist in acetone. The final setup is fixed by a surrounding polyoxymethylene (POM) chasing.

The measurement electronics consist of a custom-made spectrum analyzer scanning frequencies between 1,300 Hz to 31,300 Hz. A frequency of 1,300 Hz was previously evaluated to be most sensitive tested frequency to detect changes in impedance signal over time and was therefore used for standard data analysis.

For amperometric monitoring of the biofilm, the counter electrodes of the impedance electrodes are used as charge-collectors. For this measurement, a reference electrode is required. The latter consists of a custom-made POM casing which can be reused. It is filled with 0.05 M potassium hexacyanoferrate (Fe(II)/Fe(III)) electrolyte dissolved in phosphate buffered saline (PBS) and uses a graphite rod (Conrad Electronics SE, Germany) as cathode. The reference electrode is placed on top of the flow cells and thus in fluidic contact with the microfluidic flow channels. Nafion beads (NR50 7–9 mesh, Sigma-Aldrich, Munich, Germany) are used as proton exchange membranes (PEM) between the measurement cell and the reference electrode´s chamber (Figs. 1 and 2).

In order to obtain biofilm data with minimized environmentally caused oscillations (due to, e.g., temperature changes) bacteria-free fluidic reference channels were incorporated and measurements performed in a differential manner. For this, values acquired from bacteria-inoculated flow channels (termed “measurement channels”) were subtracted from bacteria-free flow channels (termed “reference channels”). The differential values were normalized to the first value of the experiment and thus the offset set to 0.

### Biofilm formation

The used strain collection consisted of environmental, clinical, and wastewater isolates of the species *Pseudomonas aeruginosa* and *Stenotrophomonas maltophilia*. Additional strains from bacterial type collections were included. A list of the used bacterial strains and sources can be found in S1 Table.

Bacteria were grown in Brain Heart Infusion (BHI) 1:4 (v/v) diluted medium (Merck, Darmstadt, Germany) overnight at room temperature on a reciprocate shaker. The density of the bacterial suspensions was adjusted for inoculum to the following cell numbers. For screening of biofilm forming capacity of bacterial strains an optical density (OD; 600 nm) of 0.5 (∼5x10^8^ cells/mL) was used. Alternatively, different cell densities ranging from 10 to 10^4^ bacteria/mL were prepared for sensitivity evaluation of the biosensor. Prior to each experiment the system was sterilized by pumping 70% (v/v) ethanol through the whole setup. The measurement channels, containing bacteria suspensions, were initially probed for 3 h with bacterial inoculum and the bacteria free reference channel were probed with sterile BHI 1:4 medium. After 3 h, all channels were switched to continuous medium feed at a flow rate of 100 μL/min (equals 2.5-times medium replacement/min) using manually actuated three-way cock valves. Bubble-traps were inserted between the medium connection and the channels to avoid air trapping inside of the system. The fluidic setup is depicted in S1 Fig.

Biofilm destabilization experiments were performed using commercially available detergent 1% (w/v) Tergazyme (Sigma-Aldrich). For biofilm inactivation the commonly used disinfectant Sterillium (BODE Chemie GmbH, Hamburg, Germany) was used. Preformed biofilms (2–3 days old, depending on the strains) were exposed to the different solutions between 1 h and 1.5 h at a flow rate of 100 μL/min at room temperature.

### Biofilm staining and exoenzymatic activity analyses

Staining of biofilms was performed at the end of the experiments according to manufacturer instructions using LIVE/DEAD BacLight Bacterial Viability Kit (Invitrogen, Karlsruhe, Germany). The substrates with the biofilm-covered electrodes were removed from the system by disassembling and planktonic cells were eliminated by a washing step with sterile buffer (5 mM magnesium acetate, 10 mM Tris-base, pH 8, both Sigma-Aldrich). Finally electrodes were stained for 15 min simultaneously using 5 μM SYTO9 and 15 μM propidium iodide and washed again. Images were acquired with an Axioplan2 imaging system (Zeiss, Oberkochen, Germany) at 100-fold magnification. Digital images were recorded at the same light-intensities using a Zeiss AxioCamMRm camera with AxioVision 4.6 software. For correlation experiments the acquired images were analyzed for their fluorescence intensities (green fluorescence for living bacteria and red fluorescence for dead bacteria) using ImageJ software and a minimum of 8 complete circular electrodes. The numerical values recorded are the sum of the gray values divided by area, wherein gray = (red + green)/2 (according to ImageJ user guide IJ 1.46r).

Crystal violet (CV) staining was performed to study the biofilm behavior of the different bacterial strains in a static system in comparison to the microfluidic approaches using the online biofilm monitoring. For doing so, 200 μL/well of bacteria suspensions were seeded in a 96-well plate (Nunc, Roskilde, Denmark) and incubated at room temperature on a reciprocate shaker at 50 rpm. For static biofilm formation an OD of 0.1 was used and wells were washed after 18 h with 200 μL water to remove planktonic cells. The attached biofilm biomass was stained afterwards for 10 min with 100 μL 0.05% (v/v) CV solution (Sigma-Aldrich) modified according to O'Toole and Kolter [23]. After washing twice, CV bound to bacterial cells was dissolved in 33% (v/v) acetic acid. Absorption was measured at 560 nm using a Mutiscan MS photometer (Labsystems, Helsinki, Finnland).

Exoenzymatic activity was determined by fluorescein diacetate (FDA) assay according to [24]. Esterases are extracellular enzymes of metabolically active bacteria. They are able to cleave fluorescein diacetate into fluorescein and two acetate molecules. The resulting increase in fluorescence is measured at 485 nm excitation wavelength and 538 nm emission wavelength (Fluoroscan Ascent, Labsystems). For the assay 10 μL of 1mM FDA solution (Sigma-Aldrich) dissolved in a ethylenglycol-monomethylether/water solution was incubated together with 200 μL of sterile filtrated outflow of the respective microfluidic flow cell for 1 h in a black 96-well plate (Nunc). After pH-adjustment with HEPES-buffer (0.1 M, pH 7.5), fluorescence intensities of the samples were measured. 0–10 μM fluorescein-sodium (Sigma-Aldrich) was used as standard for calibration.

### Gene expression analysis

Expression of three different flagellar and fimbrial genes (*flgD*, *flgE* and *cupA1*) playing an important role in attachment and start of biofilm formation of *P*. *aeruginosa* were analyzed in biofilms grown inside the sensor platform. For this single channels were stopped at different points in time (1 h, 4 h and 24 h after start of the experiment). RNAprotect reagent (RNeasy Protect Bacteria Mini Kit, Qiagen, Hilden, Germany) was added to the channels and incubated at room temperature for 10 min in order to stabilize the RNA. Afterwards, channels were rinsed with TE-buffer (10 mM TrisCl, 1 mM EDTA, pH 8.0) and cells were lyzed by incubation for 30 min with 5 mg/mL lysozyme (Sigma-Aldrich). The extract was pumped out of the channels mechanically. The final content was used for RNA isolation according to manufacturer protocol followed by DNAse digestion using turbo DNA-free kit (Invitrogen). Purity of the samples from DNA was confirmed by using an aliquot for PCR and subsequently agarose gel electrophoresis. Concentration of pure RNA was evaluated using Nanodrop analyzer (Spectrophotometer NP 1000, Peqlab, Erlangen, Germany). 9.62 μL of RNA were used as template for reverse transcription reaction. Reverse transcription into cDNA was performed according to manufacturer instructions (Taqman reverse transcription kit, Live Technologies, Darmstadt, Germany) [25].

Finally gene expression analysis was performed according to previous studies [26] with slight modifications. For this, cDNA was used as template for PCR reaction with specific primers (S2 Table) and the final PCR product was separated by PAGE. Gels were stained for 15 min using GelRed (Biotium, Hayward, US). Bands were quantified densitometrically using Biomedical Light Units (BLU) mode using LumiImager (Roche, Mannheim, Germany).

## Results

### Sensor validation

**Fig 3:**
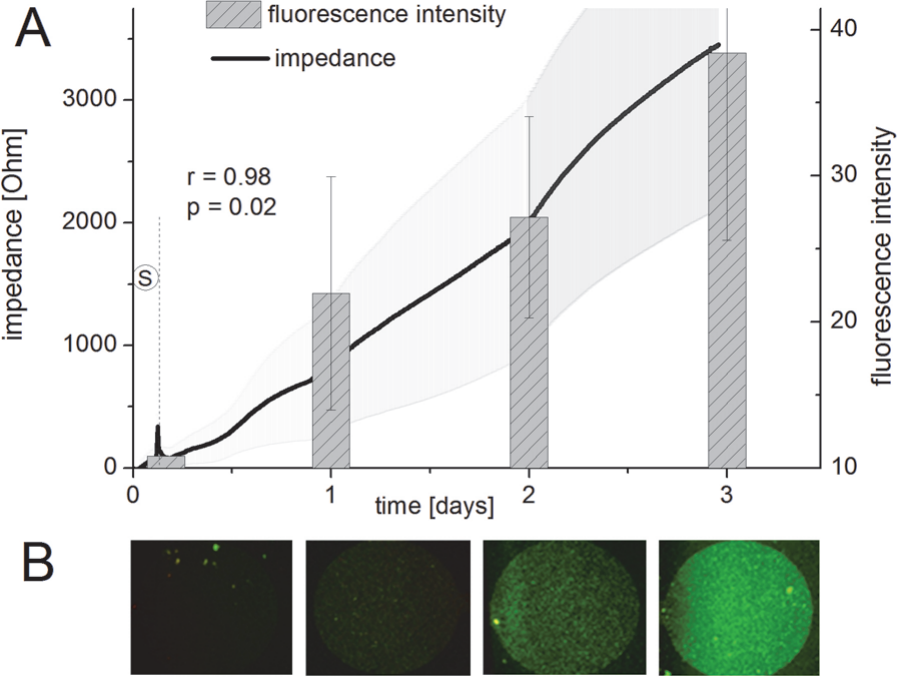
Correlation of impedance signal with fluorescence microscopy. Intensity of fluorescence images of the stained biofilm on the electrodes at different points in time (0.2 d = directly after seeding phase (S), 1 d, 2 d and 3 d) (B) were compared to the impedance signal (A). *P*. *aeruginosa* strain PA 49 in BHI 1:4 medium was used for the experiments. Values indicate means and standard deviations of 8 electrodes.

**Correlation experiments.** The developed sensor platform allows recording of biofilm thickness (overall biomass) by impedance measurement and biofilm respiratory activity by amperometric current measurement. In order to assess the correlation of this data, conventional methods of biofilm analysis were performed, in parallel and the resulting data correlated. The impedance signal, indicating the progress of attached biomass, was compared with the attached biomass detected by fluorescence staining. For this, the environmental isolate *P*. *aeruginosa* strain PA 49 was used for biofilm formation characterization over a period of 3 days in BHI 1:4 diluted medium. The modular assembly of the sensor system allowed sequential analysis at different time points (directly after seeding and 1, 2, and 3 days after seeding). Using fluorescence staining, microscopy images were recorded at identical exposure times. Afterwards the intensity of the images was measured using ImageJ software. Intensity per area was plotted against the sampling time and compared with the impedance signal progression (signal of the measurement channels with bacteria subtracted by the reference signal from channels without bacteria) (Fig. 3). Results showed that the impedimetric signal correlated well with the attached biomass measured by fluorescence staining. Intensity of the images per μm^2^ increased from 10 to 40 during 3 days, indicating gain in biofilm biomass attached to the surfaces. In total, the differential impedimetric signal (signals from measurement minus signals from reference channels) increased to more than 3500 Ω during the course of the experiments. As can be seen from Fig. 3, the fluorescence signal measured at discrete time points correlated well with the impedance signal measured. A Pearson correlation coefficient of r = 0.98 (p = 0.02) for image intensity and impedance signal strongly indicated the ability of the sensor to detect biofilm attachment over several days.

Reproducibility of the system was also tested by repeating the same assay on different days. Internal standard deviations of the differential impedance values of each run (intra-run reproducibility) ranged around 20%. For the differential signals across various runs (inter-run reproducibility) higher differences were observed, and measured signals ranged from 2000 to 4000 Ω (data for three days experiments, see S2 Fig.). Although the correlation coefficient between the curves was quite high (0.98), standard deviations between the curves were around 35%. Still, strain PA 49 was in all replicates classified in the same biofilm forming group (good biofilm former). Differences of the impedimetric signal were confirmed by microscopic image analysis of the final biofilms which differed between 17.3 and 34.5 intensity per μm^2^. In this analysis, the dependence of biofilm formation on surrounding factors like e.g. temperature becomes obvious and highlights the importance of replicates before classifying a strain. Here at least two independent assays with two replicates were performed (see section *Screening for biofilm formation capacity)*.

**Fig 4:**
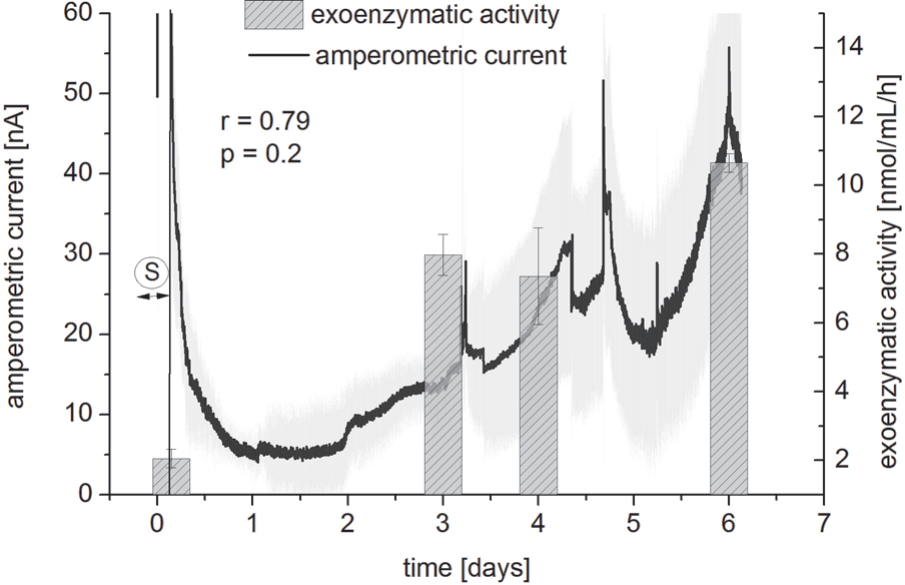
Correlation of amperometric signal with exoenzymatic activity. Amperometric signal was compared to exoenzymatic activity (esterase activity in the supernatant) of the biofilm. *P*. *aeruginosa* was grown for 6 days in BHI 1:4 medium. Samples from the outflow of the microfluidic channels were collected at different points in time and analyzed in duplicates. Seeding phase is indicated (S). Standard deviations given for amperometric current are from 4 different electrodes. r = Pearson correlation coefficient; p = significance

The amperometric activity signal was compared to the exoenzymatic esterase activity of the biofilm supernatant determined by FDA assay (Fig. 4). For this, *P*. *aeruginosa* was grown in BHI 1:4 diluted medium and the outflow from the biofilm channel was collected at different points in time (after seeding phase of 3 h, as well as after 3, 4, and 6 days). Enzymatic activity increased over this time from 2 nmol/mL/h to 10 nmol/mL/h. In parallel, a continuous increase of the amperometric current was observed. The measurement was conducted in differential mode (signals from measurement channel minus signals from reference channel). The amperometric current measured from the respiratory activity of the bacteria rose from 5 to 55 nA during the experiment. Slight differences of both curves can be observed during day 3 to day 4. During this phase, the exoenzymatic activity stayed constant, whilst the amperometric activity was still increasing. This fact might be due to the non-continuous exoenzymatic activity measurement, where fluctuations in enzyme secretion were not documented. In contrast to this approach, the amperometric current represents a continuous read-out of the respiratory activity. Considering that enzymatic activity is only a partial indicator of respiratory and metabolic activity, the same tendencies of both curves (r = 0.79, p = 0.2) indicated that the sensor reliably detected changes in the activity of the biofilm.

**Fig 5:**
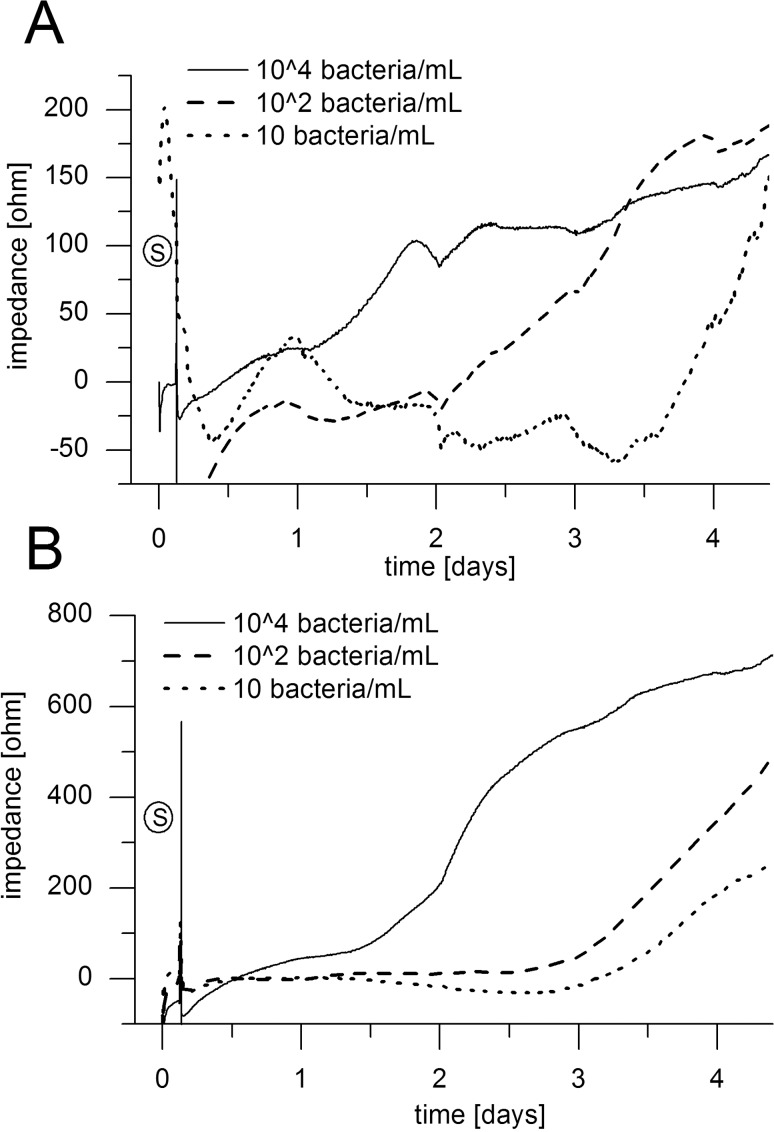
Sensor sensitivity. Sensitivity of the sensor system was tested over 4.5 days with inocula of 10, 10^2^ and 10^4^ bacteria/mL of *P*. *aeruginosa* strain PA 49 using 15 μm-gap interdigitated electrodes (A) and circular (B). Seeding phase is indicated (S).

**Sensitivity testing.** The sensitivity of the sensor was tested with different electrode designs to find the best possible resolution during all growth phases, including attachment phase at the beginning of biofilm formation as well as long-term studies of several days and weeks. Circular electrodes with 500 μm gaps between measurement and counter electrode provide reproducible detection of high number of attached bacteria and are therefore suitable for long-term studies. Interdigitated electrodes with gaps down to only 15 μm gap provide sensitive detection of only few attached bacteria. A disadvantage of narrow electrode gaps is the fast saturation with attached bacteria resulting in fast signal saturation. Exemplarily this can be seen in Fig. 5. Inoculum ranging between 10–10^4^ bacteria/mL of *P*. *aeruginosa* strain PA49 was infused to the sensor platform for 3 h and then switched to sterile medium feed. Using interdigitated electrodes for biomass detection signal onset for 10^4^ bacteria/mL can be observed after 1 day but the signal reaches a plateau phase already reached about 2 days (Fig. 5A). Using the same inoculum with circular electrodes an onset of exponential biofilm growth can be observed only after about 1.5 days (Fig. 5B). As can be seen, there is no plateau indicating that this electrode configuration does not saturate during the course of this experiment. This indicates the higher sensitivity of the interdigitated electrode design but superior long-term sensitivity of the circular electrode design. The same holds true for inocula with lower cell numbers (Fig. 5). Combinations of both electrode types within a single microfluidic channel therefore provide sensitivity throughout all biofilm development stages which is why this design was preferred during this study (see also Fig. 1C).

### Sensor application

**Fig 6:**
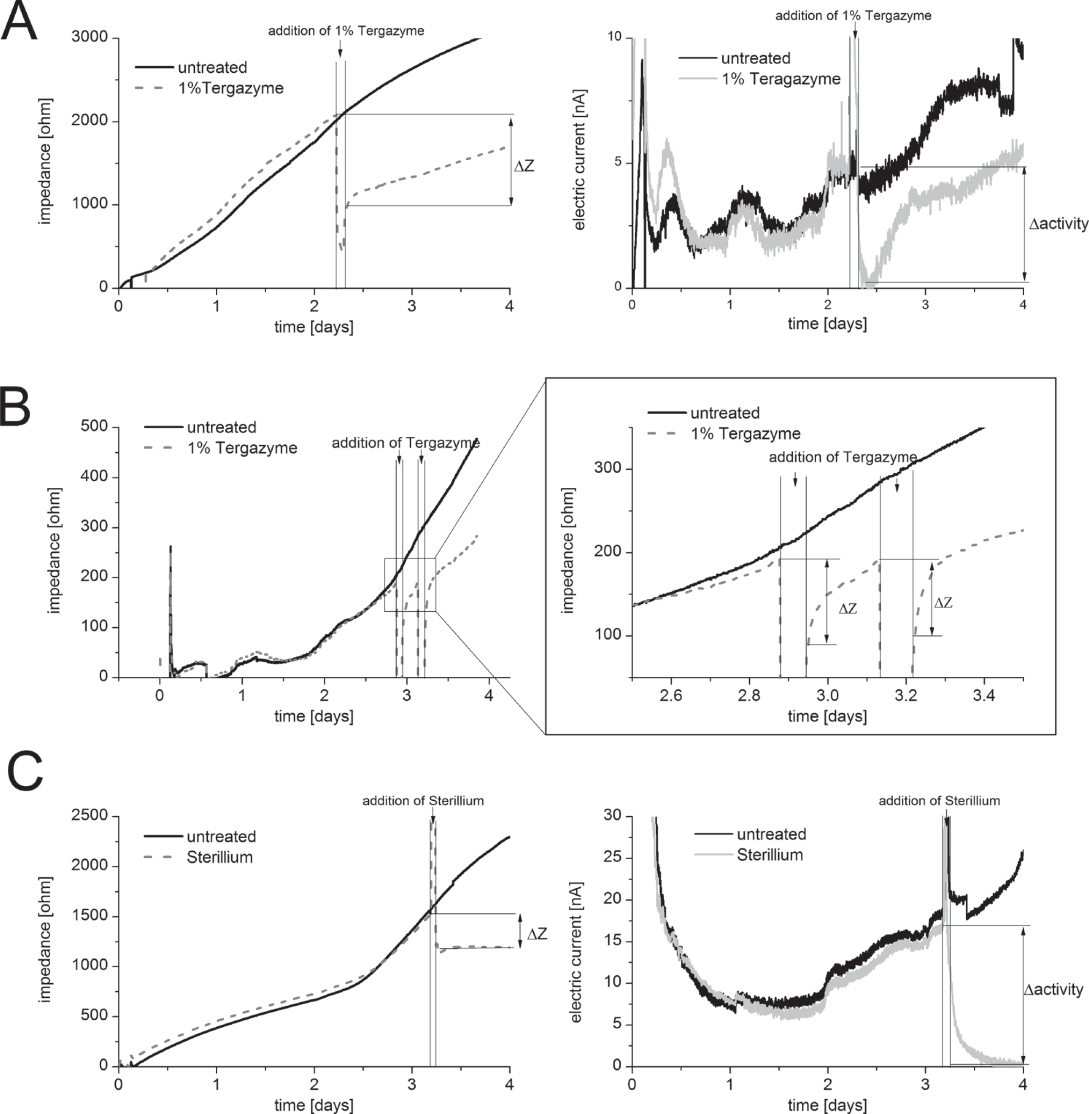
Sensor application. Biofilm destabilization and inactivation can be monitored using the sensor system. (A) Destabilization of a preformed biofilm (2 days old) of *P*. *aeruginosa* strain PA 49 which was exposed for 1.5 h to 1% Tergazyme. In comparison to an untreated biofilm a reduction of about 40% of the signal (and in correlation of the biomass) can be observed (left) in parallel also activity decreased (right). (B) Repeated destabilization on preformed biofilm (3 days old) of *P*. *aeruginosa* strain PA 14 which was exposed twice to Tergazyme for intervals of 1.5 h each. Iterated reduction in biomass can be observed. (C) Inactivation of a preformed biofilm (3 days old) of *P*. *aeruginosa* strain PA 14 which was exposed for 1 h to Sterillium. Signals of biomass (left) and activity (right) were compared between untreated and treated biofilms and are results of (at least) two independent electrodes.

**Biofilm destabilization.** For biofilm destabilization the commercial available cleaning detergent Tergazyme was applied as 1% (w/v) solution in water to a 2 day old biofilm of *P*. *aeruginosa* strain PA 49 for 1.5 h in the sensor system (Fig. 6A, left). Compared to the untreated biofilm a decrease from 2200 Ω to 1300 Ω in differential impedimetric signal was observed after treatment. This 40% signal reduction (indicating reduction in biomass) was followed by a re-rising after switching back to feeding medium without detergent. After two further days of incubation with medium, the attached biomass reached approximately the starting level before treatment. The biofilm respiratory activity, monitored by amperometry, showed a decrease in signal after addition of Tergazyme, but rose in parallel to the increasing biomass after switching back to medium (Fig. 6A, right). The decrease in both impedance and amperometry between untreated and treated biofilms was due to a reduction of biomass. This indicated that Tergazyme had a destabilizing effect (resulting in detachment of the biofilm) but no biocidal effect. The measured activity recovered completely on day 4 (5 nA before and after) whereas the total biomass was still lower than before treatment (2000 Ω before and 1600 Ω on day 4). This might suggest that the regrowing biofilm is reacting to the treatment by a stronger respiratory activity which might be part of bacterial stress responses in comparison to the untreated condition before. Repeated incubation with 1% Tergazyme resulted in repeated impedance signal drops indicating iterated reduction in biofilm biomass (Fig. 6B). Amperometric activity signal followed the same trend as the impedance signal during this prolonged experiment (data not shown). It became obvious that the biomass recovery occurred in a faster manner in case of the second treatment. These results could be discussed as a kind of biofilm adaptation to repeated external stimuli.

**Biofilm inactivation.** For biofilm inactivation Sterillium, which is a commonly used disinfectant, was tested by application to a 3 day old biofilm of *P*. *aeruginosa* strain PA 14 for 1 h in the sensor system. Increases in signal during the first 3 days of medium supply without any treatment indicated increases in attached biomass on the top of the sensor electrodes (Fig. 6C, left). After treatment with Sterillium a slight decrease in biomass, correlating to a decrease in impedance signal from 1500 Ω to 1200 Ω, was observed. Still, most of the biofilm remained attached to the electrode. No further increase in impedance signal was observed after another day of feeding with medium. Rising signals of the untreated channel up to 2400 Ω indicated further gain of biomass without treatment. These findings were supported by the strong decrease in amperometric activity from 18 nA down to 0 nA for the treated biofilm (Fig. 6C, right) indicating absence of respiratory-active bacteria by the biocidal impact of Sterillium. Even after one further day of incubation with sterile medium no regeneration of the remaining biomass was observed for the Sterillium treated biofilm. In contrast, the untreated active biofilm showed a rising amperometric signal up to 25 nA. These results indicated that Sterillium kills the biofilm but it does not reliably remove it from the surface indicated by only a slight decrease in the impedance signal. It can be reasoned that these signal drop is rather due to the removal of loosely attached biomass from the surface of the biofilm. Residual biomass after inactivation holds the risk to have still quiescent persister cells present in the biofilm, which can only be detected if these cells regain a respiratory active lifestyle.

**Fig 7:**
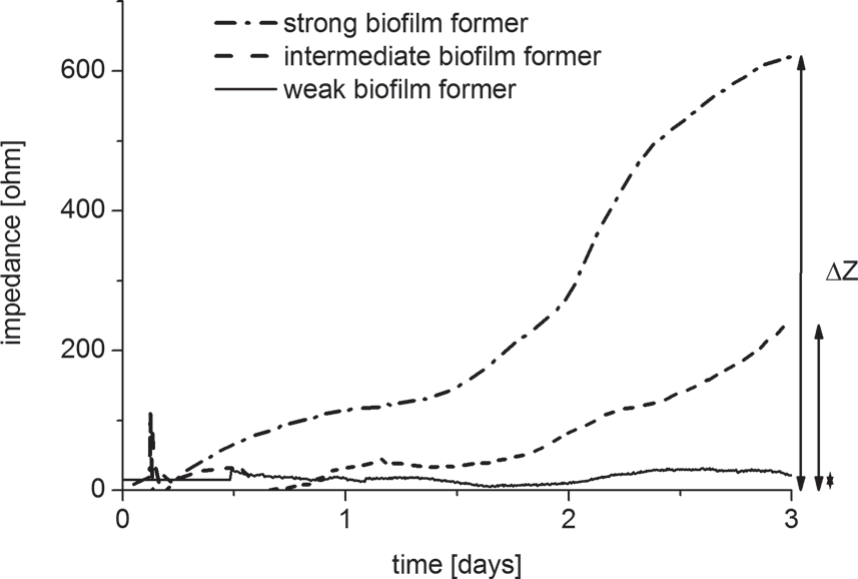
Exemplary biofilm biomass dynamics. Impedance curves of 3 different isolates of *P*. *aeruginosa* and *S*. *maltophilia* are shown, which are categorized as strong, intermediate and weak biofilm formers according to their increase in biomass (ΔZ). Biofilms were grown for 3 days in BHI 1:4 medium in the sensor system.

**Screening for biofilm formation capacity.** A strain collection of 26 isolates of *P*. *aeruginosa* and 47 isolates of *S*. *maltophilia* was screened for their biofilm formation capacities (S1 Table). For this, biofilm growth was monitored over 3 days. Afterwards the strains were classified by their increase in impedance into three groups (Fig. 7). Strains with an increase in impedance signal below 100 Ω were classified as weak biofilm formers, strains with an increase between 100–200 Ω as intermediate biofilm former, and an increase over 200 Ω indicated strong biofilm formers. Overall, 19 strains (9 *P*. *aeruginosa*, 10 *S*. *maltophilia*) were identified with a high biofilm formation potential, 18 strains (5 *P*. *aeruginosa*, 13 *S*. *maltophilia*) had intermediate potential and 36 strains (12 *P*. *aeruginosa*, 24 *S*. *maltophilia*) were weak biofilm formers (Table 1). All detailed data are listed in S1 Table. Additionally Gram-positive type-strains of *S*. *aureus* and *B*. *subtilis* were tested for biofilm formation in the sensor platform resulting in good (*S*.*aureus* DSM 20231) and intermediate (*B*. *subtilis* DSM10) biofilm forming capacity (data not shown).

**Table 1:** Classification of isolates according to biofilm formation capacity.

Biofilm forming capacity	Increase in impedance (ΔZ)	PA (26 isolates)	SM (47 isolates)
+	>200 Ω	12	10
ᴓ	100–200 Ω	5	13
-	<100 Ω	9	24
Correlation fluidic vs. static		r* = 0.34	r* = 0.20

* Pearson correlation coefficient

PA = *P*. *aeruginosa*

SM = *S*. *maltophilia*

Comparing the biofilm formation capacity of *P*. *aeruginosa* and *S*. *maltophilia* a high variability among the different isolates was found. Both facultative pathogens exhibited strains with high but also strains with very low biofilm formation ability. Furthermore, biofilm forming capacities were not dependent on the origin of isolation of the strains (S1 Table). No significant differences were observed among environmental and clinical patient’s isolates. A higher portion of good biofilm forming strains was found for *P*. *aeruginosa* under the applied conditions. Typical biofilm development stages could be identified when looking at the biomass signal (impedance). Focusing on the strong biofilm former (Fig. 7) the induction phase of biofilm formation was observed for the first 1.5 days transitioning to an exponential biofilm growth phase. After about 2.5 days a stationary biofilm phase was reached. Further, dispersal stages might be seen after additional days of incubation, but were deemed beyond the scope of this work.

In addition to the experiments using the microfluidic electrochemical sensor platform, biofilm formation capacity was determined in a conventional static 96-well format. Strains were sorted by their absorption of CV ranging between 0–1.0 as weak, 1.0–1.5 as average, and above 1.5 as good biofilm formers. Classification identified 22 weak, 31 intermediate and 22 good biofilm formers. Again, high strain specificities became obvious (S1 Table). Comparing the biofilm formation potential of strains formed in the microfluidic impedance sensor to biofilms formed in static 96-well plates differences could be observed. Low Pearson correlation between the results of both assays (r = 0.26 for *P*. *aeruginosa* and r = 0.20 for *S*. *maltophilia*; Table 1) indicated missing accordance between different biofilm culture conditions and showed the limitation of conventional static biomass determination methods. Differences in applied shear stress, the fluidic conditions or the different availability of nutrients might be factors influencing biofilm formation in the two applied setups and thus account for the reported differences.

**Fig 8:**
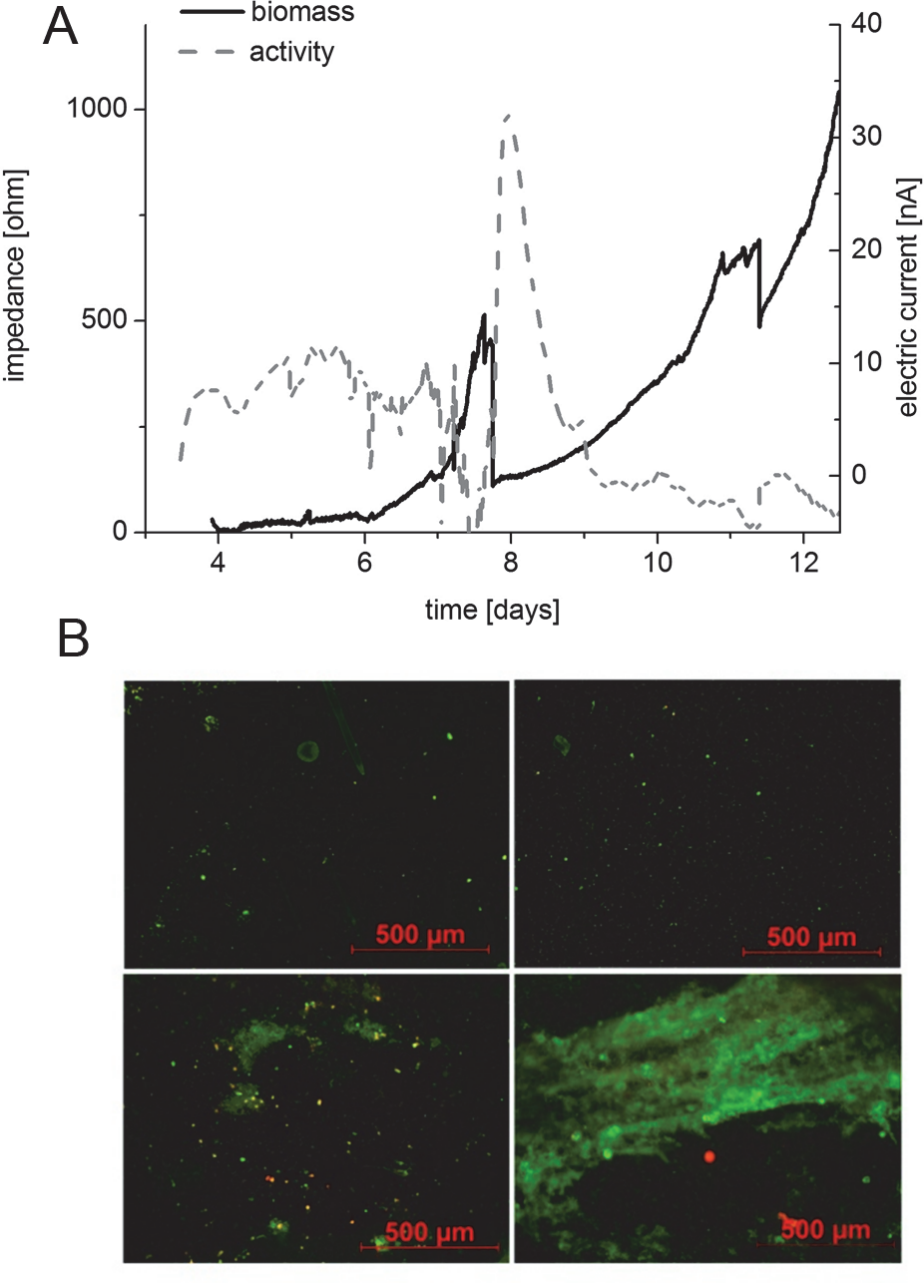
Environmental biofilm. Probing of the biosensor system with outflow of a wastewater treatment plant which was used as inoculum for the system. After inoculation for 3 days the valves were switched to sterilized outflow of the sewage plant as further nutrient source. (A) Biofilm biomass was recorded by impedance and increased after an initiation phase after 6 days. Activity of the biofilm was monitored by changes in the produced amperometric current and stayed constant during the experiment. Seeding phase is not shown because of missing of an appropriate reference channel. (B) Live dead images of the electrodes were acquired after 1 d, 2 d, 3 d and at the end of the experiment.

**Environmental biofilm formation.** Additionally, the sensor system was tested for its suitability for monitoring of complex environmental biofilms. Therefore, the sensor was inoculated with native outflow of a sewage plant for 3 days to allow attachment of a mixed species population. Afterwards, nutrient supply was switched to sterilized outflow of the sewage plant. Biofilm formation and activity were monitored over a time period of 12 days (Fig. 8A). Biofilm formation was detected after 5 days and increased during the course of the experiment to 1100 Ω. Due to the high oscillation of the baselines during inoculation phase biofilm dynamics were documented after 3 days when a primary biofilm was established. To prove the dynamics during these first days fluorescence images from Live/Dead staining were acquired at different points in time (1d, 2d, 3d, and at the end of the experiment) which confirmed the gain in biomass (Fig. 8B). The amperometric readout stayed almost constant over the first 8 days at about 10 nA. A maximum is reached at day 8 followed by a slight decrease in activity. One reason for these observations might be the low overall respiratory activity known from environmental mixed population consortia. Also the low nutrient content provided by the feeding supply might be a reason for the low activity over the course of the experiment even though biomass was increasing. Still, these results reveal the great potential of the sensor system to monitor biofilm formation even in complex microbial systems.

**Fig 9:**
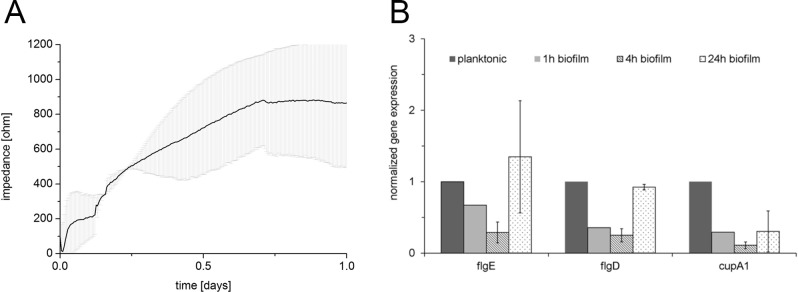
Gene expression analysis. Biofilm biomass dynamics of *P*. *aeruginosa* strain PA 57 were recorded for 24 h in parallel to gene expression (A). Data results of at least two independent assays performed in duplicates. Flagellar and fimbrial genes *flgE*, *flgD* and *cupA1* were analyzed for their expression in the planktonic state of *P*. *aeruginosa* strain PA 57, and in 1 h, 4 h and 24 h biofilms (B). Individual expressions were normalized to *rpoD* and scaled to the planktonic state. Replicates result from at least two independent experiments.

**Gene expression analysis.** Expression of three different flagellar and fimbrial genes (*flgE*, *flgD*, and *cupA1*) playing an important role in attachment and initiation of biofilm formation of *P*. *aeruginosa* was analyzed in biofilms grown inside the sensor system. For this, single channels were stopped at different points in time. Cells were lysed directly inside the channels and the extracted content was used for RNA isolation followed by a reverse transcription reaction. Finally, cDNA was amplified by PCR and the amplicons were separated by PAGE (S3 Fig.). The band intensity was densitometrically evaluated using a LumiImager working station. Data was normalized to the expression of the housekeeping gene *rpoD* and scaled to the planktonic expression levels (Fig. 9). Finally, for the *P*. *aeruginosa* strain PA 57, which was classified before as good biofilm former (S1 Table), a high gene expression of all investigated attachment genes was observed in the planktonic state. During attachment to the surface a decrease in activity of this genes could be seen (1 h and 4 h after start of inoculation), indicating the importance of their activity for initial attachment already within the first minutes. A down regulation in gene expression after attachment suggested that motility has now adverse effect on irreversible attachment and biofilm growth. The decrease in activity was followed by a rerise in flagellar and fimbrial gene expression after 24 h when the biofilm growth reached a plateau phase (Fig. 9). This rerise in gene expression of the investigated appendages suggests their importance in different stages of biofilm formation including detachment during biofilm maturation. Finally, the data demonstrated the ability of the system for gene expression analysis and thereby enables detection and measurement of molecular responses of biofilm bacteria within the sensor system.

## Discussion

The presented electrochemical sensor system represents a novel tool for online biofilm monitoring in 12 fluidically independent microfluidic channels, in parallel. The combination of amperometric activity measurement and EIS allows parallel and time-resolved determination of biofilm viability and estimation of attached biofilm biomass. Changes in the impedimetric signal during the attachment phase as well as signals obtained from experiments running over several days were highly correlating to established biofilm staining assays. The respiratory activity of the biofilm measured by amperometry exhibited the same characteristics as determined for the exoenzymatic activities using well-known assays indicating the reliability of the method for measuring the biofilm activity online. Compared to other established sensor techniques, this is the first combination of these two electrochemical methods in a microfluidic assay allowing biofilm analysis with capability for large-scale screenings. Especially the possibility for a modular system expansion with up to four connectable 12-flow channel units will enable high throughput analysis.

Application of different electrode designs facilitated sensitive detection during both the initial biofilm attachment phase as well as during long-term experiments, which allows monitoring the biofilm dynamics over several days and weeks. As bacterial attachment and thereby formation of a conditioning film are the initial phases of biofilm formation and indicate the starting of biofouling, it is important to have sensitive sensors for early-on detection and monitoring [27]. On the other hand, slowly growing populations such as biofilms consisting of oligotrophia, require monitoring for longer periods of time ranging up to even weeks or months. Depending on the problem at hand, the electrode design of this sensor system can be easily adapted by using different interdigitated and circular electrodes designs, which allows fine-tuning of the resolution for biofilm detection and monitoring.

The suitability of this system was demonstrated for different bacteria species as well as for complex microbial biofilms of sewage plant outflow. In extension, its application for a broad range of liquids was tested with conductivities ranging from 700 μS/cm to 19 mS/cm (data not shown). The system was also successfully applied for the characterization of strain collections regarding their biofilm forming potential, highlighting differences in biofilm development stages and processes among the isolates. Typical biofouling stages including induction, exponential growth, and plateau phase were recorded for different strains. Thereby differences in the attached biofilm biomasses as well as time-dependent onset of the biofilm maturation processes could be observed between the isolates. Comparison with static assays revealed the dependence of biofilm formation on the surrounding fluidic conditions. The importance of using appropriate shear conditions is in accordance with the finding of a study by Buckingham-Meyer et al. [28]. In that study a lower efficiency of disinfectants against biofilms grown under fluidic conditions in contrast to static biofilms was observed, supporting the necessity for a fluidic setup for biofilm studies including development of new cleaning strategies.

Besides fluidic factors, strain-specific motility factors are also supposed to play a key role in biofilm formation of *P*. *aeruginosa* [23]. The flagellum is needed to approach a surface and for surface attachment [23,29]. In this study, flagellar genes were found to be highly expressed in the planktonic state of the chosen good biofilm forming *P*. *aeruginosa* strain PA 57. In the early stages of biofilm formation (1 h—4 h) the flagellar gene expression was low but increased again after 24 h, when the biofilm reached a biomass plateau phase. The same is true for *cupA1* gene. The *cupA* gene cluster belongs to a class of newly discovered extracellular appendices and promotes attachment and adherence [30]. *CupA* was even supposed to be more important in early attachment phase of *P*. *aeruginosa* than expression of type IV pili [30]. The observed expression dynamics of the investigated genes therefore confirms these findings. For gaining deeper insights into biofilm dynamics, online monitoring alone is not sufficient—the associated gene expression must also be taken into consideration. Therefore, better insights in the expression of attachment factors in this early stage biofilms (up to 24 h) might give hints for biofilm dynamic prediction.

Besides strain-specific biofilm characterization and monitoring of biofilm formation, the combination of amperometry and EIS provides insights into the behavior and the fitness of attached biomass important for monitoring of biofilm manipulation processes. Recently applied cleaning strategies of biofouling processes are mainly based on inactivation or disinfection of biofilms. Reliable real-time monitoring of these processes is often missing, leading to undetected remaining biomass serving as basis of regrowth on the surfaces [11,31]. Screening of different cleaning agents and parameters could help optimizing anti-biofilm strategies. Many different biofilm cleaning strategies besides disinfection were applied recently. Biofilm destabilization was found to be a promising approach in biofilm manipulation [7]. Fast and complete removal of biofouling on surfaces is required in many technical systems such as, e.g., pipelines in the food industry, preventing the recovery of injured but still attached biofilms. Destabilizing of the biofilm surrounding EPS renders the biofilm structure vulnerable allowing more effective penetration of cleaning agents into the biofilm. Exemplary, different EPS degrading enzymes like DispersinB [32] are reported to induce biofilm dispersal or destabilization in various species. Also D-amino acids [33] and silver ions [34] have been shown to play a role in biofilm dispersal and might be suitable for new cleaning approaches. Additional commercially available substances like Tergazyme promise great cleaning opportunities against biofouling—a fact that we could conveniently confirm during this work using the biosensor platform. Recently, combinations of D-amino acids and antibiotics showed 2 log reduction in biofilm bacteria in contrast to single use [35]. In consequence, further screening of so far unknown biofilm modulating substances should combine both biofilm removal and inactivation in order to achieve effective cleaning strategies. Using the screening potential offered by the sensor platform presented in this paper it is now possible to screen for such combinations with the aim of establishing new cleaning strategies. Here, the monitoring of residual persister cells will be primary focus, which can be detected conveniently by monitoring the remaining biomass and regrowth identifiably directly by increases of both sensor signals.

Further extension of the application spectrum will be the combination of monitoring biofilm dynamics and subsequent transcriptome and metagenome analysis. Especially in case of mixed population biofilms changes in population composition due to manipulation strategies might be traceable. The combination of biofilm monitoring and metagenomics would thereby allow a risk evaluation after cleaning processes wherein a potential selection of a subpopulation might emerge. An integration of a modified sensor system setup into technical application systems might be a next key step in achieving better understanding and thereby control of biofouling.

## Supporting Information

S1 Fig Schematic drawing of the fluidic setup for screening experiments. During seeding phase bacterial inoculum is pumped by a tubing pump via three-way cocks valves into the measurement channels whilst the reference channels are continuously fed with sterile medium. After the seeding phase, the valves were switch to sterile medium which was fed into all channels. Bubble traps were placed behind the pump in order to avoid air inflow into the system. Two independent channels were connected to include replicates for all signals.
(TIF)

S2 Fig Sensor reproducibility. Impedance signals of the same bacterial strain (*P*. *aeruginosa* strain PA 49 in BHI 1:4 medium) from different runs are displayed in the figure (inter-run reproducibility). Standard deviations of the electrodes within the same run (at least 3 replicates) are indicated in the graph (intra-run reproducibility). Live/Dead stained electrodes at the end of the experiment and intensity analysis confirm the different signal outputs. Fluorescence intensity of the images ranged between 17.3 (A), 34.5 (B) and 22.8 (C).
(TIF)

S3 Fig Gene expression analysis by PAGE. RNA was isolated from a biofilm of *P*. *aeruginosa* strain PA 57. PCR products of cDNA amplification were separated according to their product sizes (rpoD 178 bp, flgE 144 bp, flgD 120 bp, cupA1 85 bp).
(TIF)

S1 Table Bacterial isolates. Sources and references of the used strains are listed and strains were classified by their increase in impedance signal after 3 days in the sensor platform. Biofilm formation in a microtiter plate was compared to the microfluidic system. Impedance assay was performed in duplicates and at least repeated twice. Crystal violet (CV) assays were performed in quadruplicates and at least repeated twice.
(DOCX)

S2 Table Sequences of primers used in this study. (DOCX)

## References

[1] Szewzyk U, Szewzyk R, Manz W, Schleifer KH (2000) Microbiological safety of drinking water. Annual review of microbiology 54: 81–127. 11018125 10.1146/annurev.micro.54.1.81

[2] Qian P, Zhou X, He H, Xu Y (2013) Antifouling method and composition using chromen-4-one derivatives. Google Patents.

[3] Petrova OE, Sauer K (2012) Sticky situations: key components that control bacterial surface attachment. Journal of bacteriology 194: 2413–2425. 10.1128/JB.00003-12 22389478 PMC3347170

[4] Mandlik A, Swierczynski A, Das A, Ton-That H (2008) Pili in Gram-positive bacteria: assembly, involvement in colonization and biofilm development. Trends in microbiology 16: 33–40. 18083568 10.1016/j.tim.2007.10.010 PMC2841691

[5] Flemming HC, Neu TR, Wozniak DJ (2007) The EPS matrix: the "house of biofilm cells". Journal of bacteriology 189: 7945–7947. 17675377 10.1128/JB.00858-07 PMC2168682

[6] Stewart PS (2002) Mechanisms of antibiotic resistance in bacterial biofilms. International journal of medical microbiology: IJMM 292: 107–113. 12195733 10.1078/1438-4221-00196

[7] Flemming H-C (2011) Microbial Biofouling: Unsolved Problems, Insufficient Approaches, and Possible Solutions. In: Flemming H-C, Wingender J, Szewzyk U, editors. Biofilm Highlights: Springer Berlin Heidelberg pp. 81–109.

[8] Darouiche RO, Mansouri MD, Gawande PV, Madhyastha S (2009) Antimicrobial and antibiofilm efficacy of triclosan and DispersinB combination. The Journal of antimicrobial chemotherapy 64: 88–93. 10.1093/jac/dkp158 19447791

[9] Ruseska I, Robbins J, Costerton JW, Lashen ES (1982) Biocide Testing against Corrosion-Causing Oil-Field Bacteria Helps Control Plugging. Oil & Gas Journal 80: 253–&. 10.1016/j.jenvman.2015.01.014 25596922

[10] Goeres DM, Loetterle LR, Hamilton MA, Murga R, Kirby DW, et al (2005) Statistical assessment of a laboratory method for growing biofilms. Microbiology 151: 757–762. 15758222 10.1099/mic.0.27709-0

[11] Strathmann M, Mittenzwey KH, Sinn G, Papadakis W, Flemming HC (2013) Simultaneous monitoring of biofilm growth, microbial activity, and inorganic deposits on surfaces with an in situ, online, real-time, non-destructive, optical sensor. Biofouling 29: 573–583. 10.1080/08927014.2013.791287 23682638

[12] Fischer M, Wahl M, Friedrichs G (2012) Design and field application of a UV-LED based optical fiber biofilm sensor. Biosensors & bioelectronics 33: 172–178. 10.1002/adma.201404741 22265878

[13] Kirschhofer F, Rieder A, Prechtl C, Kuhl B, Sabljo K, et al (2013) Quartz crystal microbalance with dissipation coupled to on-chip MALDI-ToF mass spectrometry as a tool for characterising proteinaceous conditioning films on functionalised surfaces. Analytica chimica acta 802: 95–102. 10.1016/j.aca.2013.10.007 24176510

[14] Reipa V, Almeida J, Cole KD (2006) Long-term monitoring of biofilm growth and disinfection using a quartz crystal microbalance and reflectance measurements. Journal of microbiological methods 66: 449–459. 16580080 10.1016/j.mimet.2006.01.016

[15] Jenkins AT, ffrench-constant R, Buckling A, Clarke DJ, Jarvis K (2004) Study of the attachment of Pseudomonas aeruginosa on gold and modified gold surfaces using surface plasmon resonance. Biotechnology progress 20: 1233–1236. 15296453 10.1021/bp034367u

[16] Kim YW, Sardari SE, Meyer MT, Iliadis AA, Wu HC, et al (2012) An ALD aluminum oxide passivated Surface Acoustic Wave sensor for early biofilm detection. Sensors and Actuators B-Chemical 163: 136–145.

[17] Richter L, Stepper C, Mak A, Reinthaler A, Heer R, et al (2007) Development of a microfluidic biochip for online monitoring of fungal biofilm dynamics. Lab on a chip 7: 1723–1731. 18030393 10.1039/b708236c

[18] Paredes J, Becerro S, Arana S (2014) Label-free interdigitated microelectrode based biosensors for bacterial biofilm growth monitoring using Petri dishes. Journal of microbiological methods 100: 77–83. 10.1016/j.mimet.2014.02.022 24632516

[19] Paredes J, Becerro S, Arizti F, Aguinaga A, Del Pozo JL, et al (2012) Real time monitoring of the impedance characteristics of Staphylococcal bacterial biofilm cultures with a modified CDC reactor system. Biosensors & bioelectronics 38: 226–232. 10.1002/adma.201404741 22705402

[20] Babauta JT, Beyenal H (2013) Mass transfer studies of Geobacter sulfurreducens biofilms on rotating disk electrodes. Biotechnology and bioengineering. 10.1002/bit.25105 PMC4247833 23996084

[21] Pires L, Sachsenheimer K, Kleintschek T, Waldbaur A, Schwartz T, et al (2013) Online monitoring of biofilm growth and activity using a combined multi-channel impedimetric and amperometric sensor. Biosensors and Bioelectronics 47: 157–163. 10.1016/j.bios.2013.03.015 23570679

[22] Richardson DJ (2000) Bacterial respiration: a flexible process for a changing environment. Microbiology 146 (Pt 3): 551–571. 10746759 10.1099/00221287-146-3-551

[23] O'Toole GA, Kolter R (1998) Flagellar and twitching motility are necessary for Pseudomonas aeruginosa biofilm development. Mol Microbiol 30: 295–304. 9791175 10.1046/j.1365-2958.1998.01062.x

[24] Emtiazi F, Schwartz T, Marten SM, Krolla-Sidenstein P, Obst U (2004) Investigation of natural biofilms formed during the production of drinking water from surface water embankment filtration. Water research 38: 1197–1206. 14975653 10.1016/j.watres.2003.10.056

[25] Bruchmann J, Kirchen S, Schwartz T (2013) Sub-inhibitory concentrations of antibiotics and wastewater influencing biofilm formation and gene expression of multi-resistant Pseudomonas aeruginosa wastewater isolates. Environmental science and pollution research international 20: 3539–3549. 10.1007/s11356-013-1521-4 23392972

[26] Jungfer C, Friedrich F, Varela Villarreal J, Brandle K, Gross HJ, et al (2013) Drinking water biofilms on copper and stainless steel exhibit specific molecular responses towards different disinfection regimes at waterworks. Biofouling 29: 891–907. 10.1080/08927014.2013.813936 23875760

[27] Busscher HJ, van der Mei HC (1997) Physico-chemical interactions in initial microbial adhesion and relevance for biofilm formation. Advances in dental research 11: 24–32. 9524439 10.1177/08959374970110011301

[28] Buckingham-Meyer K, Goeres DM, Hamilton MA (2007) Comparative evaluation of biofilm disinfectant efficacy tests. Journal of microbiological methods 70: 236–244. 17524505 10.1016/j.mimet.2007.04.010

[29] Sauer K, Camper AK, Ehrlich GD, Costerton JW, Davies DG (2002) Pseudomonas aeruginosa displays multiple phenotypes during development as a biofilm. Journal of bacteriology 184: 1140–1154. 11807075 10.1128/jb.184.4.1140-1154.2002 PMC134825

[30] Vallet I, Olson JW, Lory S, Lazdunski A, Filloux A (2001) The chaperone/usher pathways of Pseudomonas aeruginosa: identification of fimbrial gene clusters (cup) and their involvement in biofilm formation. Proceedings of the National Academy of Sciences of the United States of America 98: 6911–6916. 11381121 10.1073/pnas.111551898 PMC34452

[31] Gibson H, Taylor JH, Hall KE, Holah JT (1999) Effectiveness of cleaning techniques used in the food industry in terms of the removal of bacterial biofilms. Journal of applied microbiology 87: 41–48. 10432586 10.1046/j.1365-2672.1999.00790.x

[32] Kaplan JB (2010) Biofilm dispersal: mechanisms, clinical implications, and potential therapeutic uses. Journal of dental research 89: 205–218. 10.1177/0022034509359403 20139339 PMC3318030

[33] Kolodkin-Gal I, Romero D, Cao S, Clardy J, Kolter R, et al (2010) D-amino acids trigger biofilm disassembly. Science 328: 627–629. 10.1126/science.1188628 20431016 PMC2921573

[34] Chaw KC, Manimaran M, Tay FE (2005) Role of silver ions in destabilization of intermolecular adhesion forces measured by atomic force microscopy in Staphylococcus epidermidis biofilms. Antimicrobial Agents and Chemotherapy 49: 4853–4859. 16304145 10.1128/AAC.49.12.4853-4859.2005 PMC1315927

[35] Sanchez CJ Jr, Akers KS, Romano DR, Woodbury RL, Hardy SK, et al. (2014) D-Amino Acids Enhance the Activity of Antimicrobials Against Biofilms of Clinical Wound Isolates of Staphylococcus aureus and Pseudomonas aeruginosa. Antimicrobial Agents and Chemotherapy 10.1128/AAC.02468-14 PMC4136027 24841260

