# Supplementary figures and images for: Multi-Channel Microfluidic Biosensor Platform Applied for Online Monitoring and Screening of Biofilm Formation and Activity
Source: PLoS One. 2015 Feb 23;10(2):e0117300. doi: 10.1371/journal.pone.0117300 (PMC4338023; doi:10.1371/journal.pone.0117300)

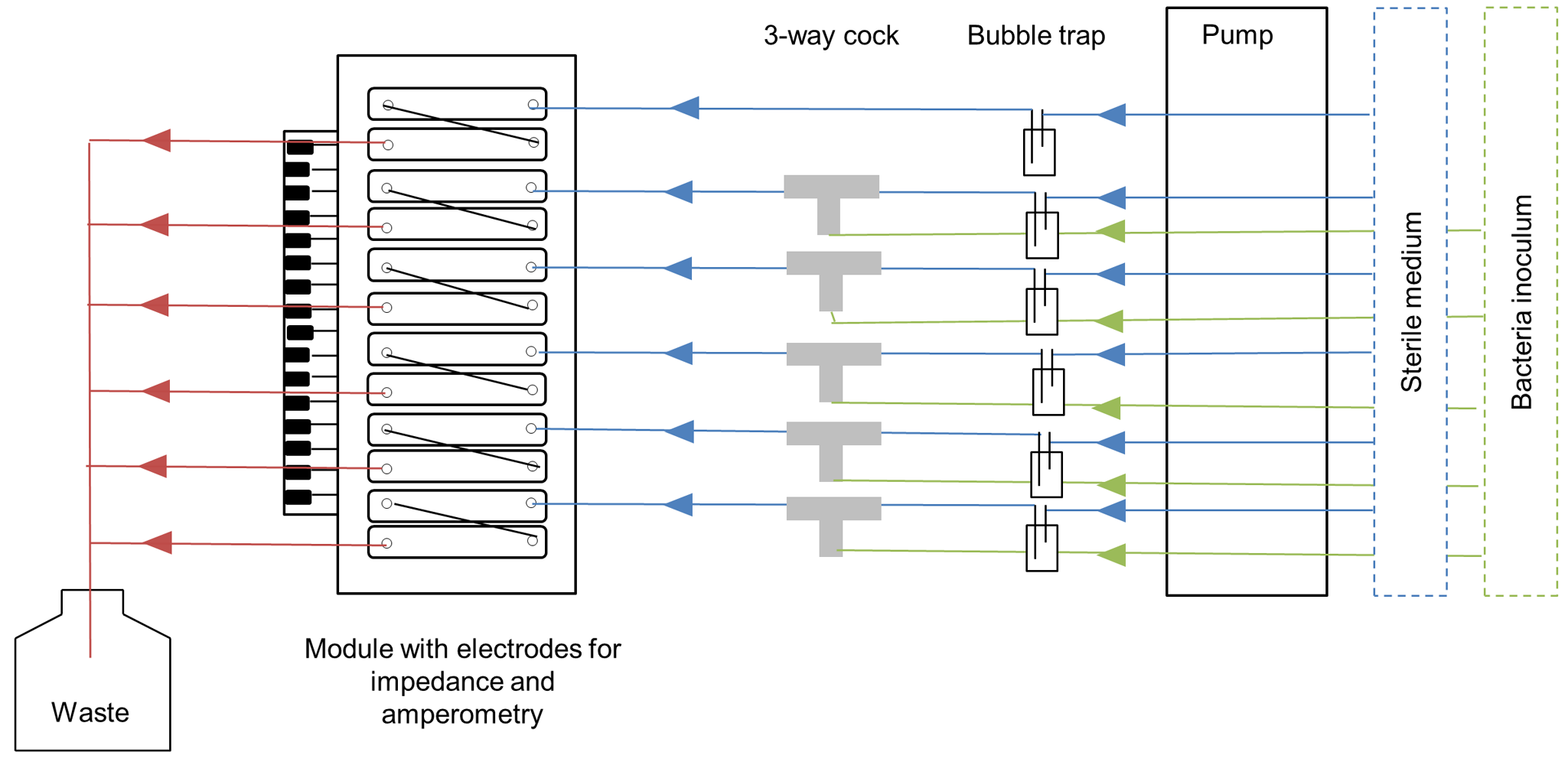

Supplement: S1 Fig — During seeding phase bacterial inoculum is pumped by a tubing pump via three-way cocks valves into the measurement channels whilst the reference channels are continuously fed with sterile medium. After the seeding phase, the valves were switch to sterile medium which was fed into all channels. Bubble traps were placed behind the pump in order to avoid air inflow into the system. Two independent channels were connected to include replicates for all signals. (TIF) [file pone.0117300.s001.tif]

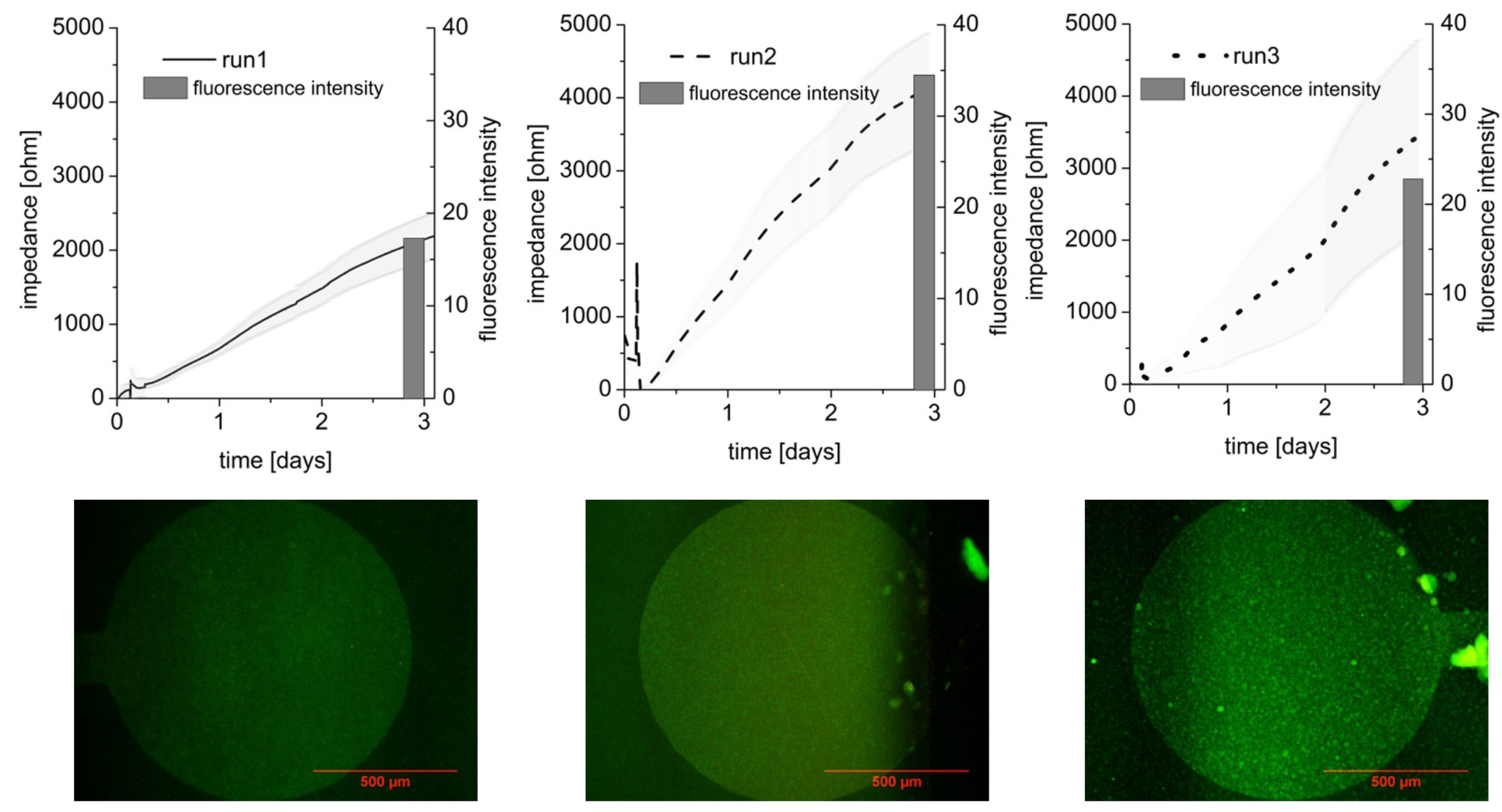

Supplement: S2 Fig — Impedance signals of the same bacterial strain (P. aeruginosa strain PA 49 in BHI 1:4 medium) from different runs are displayed in the figure (inter-run reproducibility). Standard deviations of the electrodes within the same run (at least 3 replicates) are indicated in the graph (intra-run reproducibility). Live/Dead stained electrodes at the end of the experiment and intensity analysis confirm the different signal outputs. Fluorescence intensity of the images ranged between 17.3 (A), 34.5 (B) and 22.8 (C). (TIF) [file pone.0117300.s002.tif]

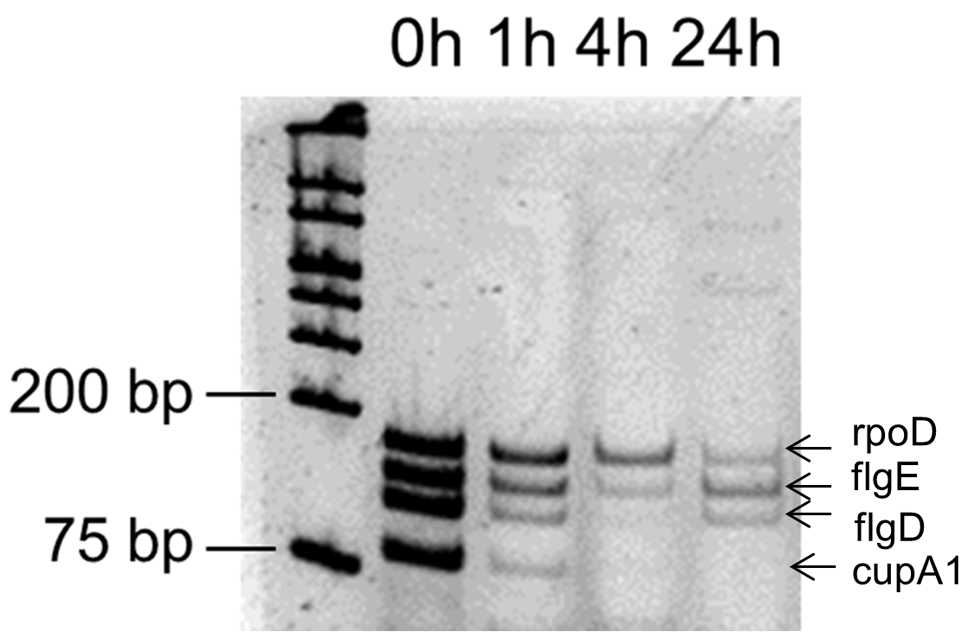

Supplement: S3 Fig — RNA was isolated from a biofilm of P. aeruginosa strain PA 57. PCR products of cDNA amplification were separated according to their product sizes (rpoD 178 bp, flgE 144 bp, flgD 120 bp, cupA1 85 bp). (TIF) [file pone.0117300.s003.tif]
