# Supplementary material for: Multi-Channel Microfluidic Biosensor Platform Applied for Online Monitoring and Screening of Biofilm Formation and Activity
Source: PLoS One. 2015 Feb 23;10(2):e0117300. doi: 10.1371/journal.pone.0117300 (PMC4338023; doi:10.1371/journal.pone.0117300)
Supplement: S1 Table — Sources and references of the used strains are listed and strains were classified by their increase in impedance signal after 3 days in the sensor platform. Biofilm formation in a microtiter plate was compared to the microfluidic system. Impedance assay was performed in duplicates and at least repeated twice. Crystal violet (CV) assays were performed in quadruplicates and at least repeated twice. (DOCX) [file pone.0117300.s004.docx]

**Table S1. Bacterial isolates.** Sources and references of the used strains are listed. Strains were classified by their increase in impedance signal after 3 days of cultivation in the biosensor platform. Biofilm formation in a microtiter plate was compared to the microfluidic system. Impedance assay was performed in duplicates and repeated at least twice. Crystal violet microtiter plate assays were performed in triplicates and repeated at least twice.

| **Strain*** | **Source** | **Reference** | **Biofilm formation potential** | |
| --- | --- | --- | --- | --- |
|  |  |  | **Fluidic setup (Impedance measurement)^#^** | **Static setup**  **(Microtiter plate)^#^** |
| **PA 01** | Wound | [[1](#_ENREF_1)] | - | -- |
| **PA 14** | Patient isolate, burn wound |  | ++ | ++ |
| **PA 154** | Clinical wastewater | [[2](#_ENREF_2)] | - | -- |
| **PA 22** | Clinical wastewater | [[2](#_ENREF_2)] | ++ | O |
| **PA 23** | Clinical wastewater | [[2](#_ENREF_2)] | - | -- |
| **PA 236** | Clinical wastewater | [3] | -- | + |
| **PA 253** | Sensitive reference strain from wastewater | [[2](#_ENREF_2)] | O | + |
| **PA 30** | Waste water | [[3](#_ENREF_3)] | - | O |
| **PA 31** | Sensitive reference strain from wastewater | [[2](#_ENREF_2)] | - | O |
| **PA 41** | Waste water | [[3](#_ENREF_3)] | + | - |
| **PA 412** | Sensitive reference strain from wastewater | [[2](#_ENREF_2)] | O | ++ |
| **PA 49** | Waste water | [[2](#_ENREF_2)] | ++ | ++ |
| **PA 55** | Sewage plant | [[2](#_ENREF_2)] | ++ | O |
| **PA 56** | Sewage plant | [[3](#_ENREF_3)] | -- | O |
| **PA 57** | Sewage plant | [[3](#_ENREF_3)] | + | ++ |
| **PA 59** | Sewage plant | [[2](#_ENREF_2)] | ++ | O |
| **PA 77** | Outflow sewage plant | [[2](#_ENREF_2)] | -- | + |
| **PA 910** | Clinical waste water | [[3](#_ENREF_3)] | O | -- |
| **PA 912** | Clinical wastewater | [[2](#_ENREF_2)] | O | - |
| **PA 966** | Clinical wastewater | [[2](#_ENREF_2)] | - | - |
| **PA 967** | Clinical wastewater | [[2](#_ENREF_2)] | - | O |
| **PA 968** | Clinical wastewater | [[2](#_ENREF_2)] | + | - |
| **PA 969** | Clinical wastewater | [[2](#_ENREF_2)] | + | O |
| **PA 987** | Intake sewage plant | [[2](#_ENREF_2)] | O | + |
| **PA 990** | Intake sewage plant | [[2](#_ENREF_2)] | -- | O |
| **PA 991** | Intake sewage plant | [[2](#_ENREF_2)] | - | + |
| **SM 4.1** | Freshwater sediment | [[4](#_ENREF_4)] | + | O |
| **SM 677** | Respiratory tract | [[5](#_ENREF_5)] | - | -- |
| **SM 682** | Respiratory tract | [[5](#_ENREF_5)] | - | -- |
| **SM 683** | Respiratory tract | [[5](#_ENREF_5)] | -- | O |
| **SM 8.1** | Freshwater sediment | [[4](#_ENREF_4)] | O | -- |
| **SM c10** | Human | [[6](#_ENREF_6)] | - | O |
| **SM c11** | Human | [[6](#_ENREF_6)] | + | - |
| **SM c16** | Human | [[6](#_ENREF_6)] | + | O |
| **SM c19** | Human | [[6](#_ENREF_6)] | O | O |
| **SM c20** | Human | [[6](#_ENREF_6)] | O | O |
| **SM c5** | Human | [[6](#_ENREF_6)] | O | O |
| **SM c6** | Human | [[6](#_ENREF_6)] | - | ++ |
| **SM DSM 50170** | Oropharyngeal region of patient with cancer | [[7](#_ENREF_7)] | ++ | ++ |
| **SM e1** | Brackish lagoon | [[6](#_ENREF_6)] | O | O |
| **SM e21** | Sewage treatment plant | [[6](#_ENREF_6)] | O | O |
| **SM e22** | Sewage treatment plant | [[6](#_ENREF_6)] | -- | O |
| **SM e4** | Rhizosphere of oilseed rape | [[6](#_ENREF_6)] | + | - |
| **SM GS1** | Activated sludge | [[4](#_ENREF_4)] | - | + |
| **SM GS5** | Activated sludge | [[4](#_ENREF_4)] | - | O |
| **SM K279a** | Blood of a cancer patient |  | O | O |
| **SM KA24** | Activated sludge | [[4](#_ENREF_4)] | - | O |
| **SM KA41** | Activated sludge | [[4](#_ENREF_4)] | + | - |
| **SM KS13** | Activated sludge | [[4](#_ENREF_4)] | -- | O |
| **SM KS14** | Activated sludge | [[4](#_ENREF_4)] | -- | -- |
| **SM LMG 10853** | Sputum | [[8](#_ENREF_8)] | O | + |
| **SM LMG 10873** | Case of conjunctivitis | [[8](#_ENREF_8)] | - | - |
| **SM LMG 10874** | Human blood culture | [[8](#_ENREF_8)] | - | - |
| **SM LMG 10991** | Leg, pus | [[8](#_ENREF_8)] | + | + |
| **SM LMG 10996** | Leg, ulcer | [[8](#_ENREF_8)] | ++ | + |
| **SM LMG 11114** | Human blood culture | [[8](#_ENREF_8)] | - | + |
| **SM NA16** | Sewage plant effluent | [[4](#_ENREF_4)] | -- | + |
| **SM NA20** | Sewage plant effluent | [[4](#_ENREF_4)] | + | O |
| **SM NB12** | Activated sludge | [[4](#_ENREF_4)] | - | - |
| **SM R551-3** | Plant tissue, *Populus trichocarpa* | [[9](#_ENREF_9)] | O | + |
| **SM RA8** | Sewage plant effluent | [[4](#_ENREF_4)] | ++ | O |
| **SM SKA14** | Marine | [[10](#_ENREF_10)] | O | O |
| **SM SKK1** | Wound swap | [[4](#_ENREF_4)] | -- | - |
| **SM SKK12** | Wound swap | [[4](#_ENREF_4)] | O | O |
| **SM SKK28** | Urine | [[4](#_ENREF_4)] | - | + |
| **SM SKK3** | Respiratory tract | [[4](#_ENREF_4)] | - | O |
| **SM SKK35** | Ulcer swab | [[4](#_ENREF_4)] | - | + |
| **SM SKK38** | Urine | [[4](#_ENREF_4)] | O | O |
| **SM SKK5** | Respiratory tract | [[4](#_ENREF_4)] | - | ++ |
| **SM SKK53** | Respiratory tract | [[4](#_ENREF_4)] | - | + |
| **SM SKK55** | Respiratory tract | [[4](#_ENREF_4)] | O | O |
| **SM X434** | Water | [[4](#_ENREF_4)] | - | -- |
| **SM X968** | Water | [[4](#_ENREF_4)] | - | O |

* PA= *Pseudomonas aeruginosa ;* SM = *Stenotrophomonas maltophilia*

^#^ Classification:

increase in impedance signal: <10 Ω = --; 10-100 Ω = -; 100-200 Ω = o; 200-300 Ω = +; >300 Ω = ++

absorbance CV (560 nm): 0-0.5 = --; 0.5-1 = -; 1-1.5 = o; 1.5-2 = +; >2 = ++

**References**

1. Hancock RE, Carey AM (1979) Outer membrane of Pseudomonas aeruginosa: heat- 2-mercaptoethanol-modifiable proteins. Journal of bacteriology 140: 902-910.

2. Schwartz T, Volkmann H, Kirchen S, Kohnen W, Schon-Holz K, et al. (2006) Real-time PCR detection of Pseudomonas aeruginosa in clinical and municipal wastewater and genotyping of the ciprofloxacin-resistant isolates. FEMS microbiology ecology 57: 158-167.

3. Bruchmann J, Kirchen S, Schwartz T (2013) Sub-inhibitory concentrations of antibiotics and wastewater influencing biofilm formation and gene expression of multi-resistant Pseudomonas aeruginosa wastewater isolates. Environmental science and pollution research international 20: 3539-3549.

4. Adamek M, Overhage J, Bathe S, Winter J, Fischer R, et al. (2011) Genotyping of environmental and clinical Stenotrophomonas maltophilia isolates and their pathogenic potential. PloS one 6: e27615.

5. Kaiser S, Biehler K, Jonas D (2009) A Stenotrophomonas maltophilia multilocus sequence typing scheme for inferring population structure. Journal of bacteriology 191: 2934-2943.

6. Minkwitz A, Berg G (2001) Comparison of antifungal activities and 16S ribosomal DNA sequences of clinical and environmental isolates of Stenotrophomonas maltophilia. Journal of clinical microbiology 39: 139-145.

7. Palleroni NJ, Bradbury JF (1993) Stenotrophomonas, a new bacterial genus for Xanthomonas maltophilia (Hugh 1980) Swings et al. 1983. International journal of systematic bacteriology 43: 606-609.

8. Hauben L, Vauterin L, Moore ER, Hoste B, Swings J (1999) Genomic diversity of the genus Stenotrophomonas. International journal of systematic bacteriology 49 Pt 4: 1749-1760.

9. Taghavi S, Garafola C, Monchy S, Newman L, Hoffman A, et al. (2009) Genome survey and characterization of endophytic bacteria exhibiting a beneficial effect on growth and development of poplar trees. Applied and environmental microbiology 75: 748-757.

10. Hagstrom A, Pinhassi J, Zweifel UL (2000) Biogeographical diversity among marine bacterioplankton. Aquatic Microbial Ecology 21: 231-244.
