# Supplementary material for: Multi-Channel Microfluidic Biosensor Platform Applied for Online Monitoring and Screening of Biofilm Formation and Activity
Source: PLoS One. 2015 Feb 23;10(2):e0117300. doi: 10.1371/journal.pone.0117300 (PMC4338023; doi:10.1371/journal.pone.0117300)
Supplement: S2 Table — (DOCX) [file pone.0117300.s005.docx]

Table S2. Sequences of primers used in this study.

| **Target gene** | **Forward primer (5’-3’)** | **Reverse primer (5’-3’)** | **Amplicon size** | **Reference** |
| --- | --- | --- | --- | --- |
| rpoD | GGGCGAAGAAGGAAATGGTC | CAGGTGGCGTAGGTGGAGAA | 178 bp | [[1](#_ENREF_1)] |
| flgE | CCACACCATGAGCCAGTTCT | TTGAAGCTCATCGGCGTCTT | 144 bp | this study |
| flgD | CCGTGGTCAATCGCATCAAC | TCTGCGCCTCGAACTTGTAG | 120 bp | this study |
| cupA1 | ATTCAGCGGCGAAGTGAC | CGGTACGCTGTCGAGGAT | 85 bp | [[2](#_ENREF_2)] |

1. Savli H, Karadenizli A, Kolayli F, Gundes S, Ozbek U, et al. (2003) Expression stability of six housekeeping genes: A proposal for resistance gene quantification studies of Pseudomonas aeruginosa by real-time quantitative RT-PCR. J Med Microbiol 52: 403-408.

2. Nicastro GG, Boechat AL, Abe CM, Kaihami GH, Baldini RL (2009) Pseudomonas aeruginosa PA14 cupD transcription is activated by the RcsB response regulator, but repressed by its putative cognate sensor RcsC. FEMS Microbiology Letters 301: 115-123.
